# Influence of Renal Impairment on the Success of Reconstruction Using Microvascular Grafts—A Retrospective Study of 251 Free Flaps

**DOI:** 10.3390/jpm12101744

**Published:** 2022-10-20

**Authors:** Henriette L. Moellmann, Nadia Karnatz, Ilkan Degirmenci, Antonina Gyurova, Lorenz Sellin, Majeed Rana

**Affiliations:** 1 Department of Oral and Maxillofacial Surgery, University Hospital Duesseldorf, 40225 Duesseldorf, Germany; 2 Department of Oral, Maxillofacial and Facial Plastic Surgery, Evangelical Hospital Bethesda, 41061 Mönchengladbach, Germany; 3 Department of Nephrology, Medical Faculty, Heinrich-Heine-University, 40225 Düsseldorf, Germany

**Keywords:** renal impairment, renal failure, microvascular reconstruction, free flaps

## Abstract

Background: In head and neck surgery, reconstruction using microvascular grafts is a successful method for functional and aesthetic restoration. Due to technological advances and medical care, the number of patients with comorbidities and diseases requiring free tissue transfer has increased. To provide adequate treatment to these patients, preoperative identification of potential risk factors is essential. Methods: In this retrospective study, we investigated the impact of renal insufficiency on reconstruction in 251 microvascular grafts. Perioperative complications, failure rate, and outcomes serve as the basis for this evaluation. Results: Comparing pre- and postoperative values, there was a significant decrease in potassium and creatinine levels and a significant increase in GFR. The electrolyte changes in relation to the complication rate showed that complications were more likely to occur as potassium levels increased. As sodium levels increase, the complication rate decreases. Conclusion: A preoperative value indicative of impaired renal function, such as creatinine levels, GFR, or electrolytes, did not prove to be an individual risk factor for the occurrence of graft failure in this patient population. Nevertheless, increased renal parameters are associated with increased incidence of serious complications. Therefore, these should be considered in the indication and preoperative planning.

## 1. Introduction

Despite the presence of comorbidities, there is a need for microvascular reconstruction even in pre-diseased patients. Success rates of 95% in free tissue transfer are reported in the current literature [1,2,3,4]. Compared to local grafting, microvascular grafts are much more challenging, but lead to better results in head and neck reconstruction [5]. In addition, the microsurgical technique allows for more sophisticated and complex reconstructions [6]. Comorbidities, such as diabetes mellitus, uremia, severe atherosclerotic disease, and preoperative radiation influence postoperative complication rates after free tissue transfer [5,7,8,9,10,11,12,13]. Many patients reveal malnutrition, nicotine abuse, and previous radiation, which, in turn, complicates overall reconstruction [1,3,5,14,15]. Due to advances in technology and medical care, there has been an increase in the number of patients with these comorbidities and conditions that require free tissue transfer after cancer resection or trauma [12,14,16]. It is thus essential to identify the comorbidities as risk factors and to consider them in the indication of surgery and preoperative planning [17]. In the study at hand, we evaluated the influence of renal impairment on the success of microvascular reconstruction. Extensive surgery is one of the most common risk factors for an acute kidney injury (AKI) and may occur postoperatively [18]. During and after surgery, large volume shifts, hypotension, nephrotoxic antibiotics, and NSAIDs may impair blood flow or damage the kidneys. In elderly patients with pre-existing comorbidities, such as diabetes, chronic kidney disease (CKD) and/or heart failure, the risk of AKI is highly increased [19]. It further occurs in 7% to 18% of hospitalized patients and is a known complication in 50% to 60% of patients admitted to the intensive care unit, which, in turn, is associated with significant mortality and morbidity [20]. AKI is reversible within the first few days to weeks in many cases, yet data from several large observational and epidemiological studies indicate a strong association between AKI and subsequent chronic kidney disease (CKD) and end-stage renal disease (ESRD) [21,22]. When AKI requires renal replacement therapy (RRT), patients are more than three times as likely to develop ESRD than those not receiving RRT. Increased numbers of ESRD patients are a pervasive problem associated with quality-of-life limitations, significant costs, and economic consequences. Therefore, prevention, early detection, and prompt treatment of AKI are of great importance [23].

To validate the influence of renal function on the complication rate after free tissue transfer in the head and neck region, we retrospectively analyzed patients who received free tissue transfer for reconstruction.

## 2. Materials and Methods

This retrospective study was approved by the local ethics committee at the University of Düsseldorf, Germany (Approval number 2022–1069). The results of microvascular reconstructions between 2015 and 2020 in the Department of Oral and Maxillofacial Plastic Surgery at the University Hospital Düsseldorf were evaluated.

### 2.1. Inclusion Criteria

All patients who underwent microvascular reconstruction at the Department of Oral and Maxillofacial Surgery at the University Hospital Düsseldorf between 2015 and 2020 were included. All cases operated as primary or secondary reconstruction have been evaluated.

### 2.2. Patients’ Data Acquisition

Based on patient data, the course, the healing process, and the postoperative outcome were compared. The following patient- and graft-related data were collected from clinical documentation, surgical reports, and findings:

- Patient data (name, age, date of birth, gender)
- Preoperative (previous operations, concomitant diseases/pretreatments with possible effect on wound healing, etiology of the defect, histology of the defect, preoperative radiological findings, localization of the defect, blood parameters)
- Surgery (date, type of graft resection limits, ischemia time, duration of surgery, surgical technique, graft, complications during anastomosis)
- Inpatient stay (wound healing process, complications, length of stay)
- Postoperative course (sensitivity disorders, pain, pressure sensitivity, skin conditions, scar conditions, complications, blood parameters)

### 2.3. Statistical Analysis

The determined values of the measurements and the clinical data were statistically analyzed using jamovi (version 1.6.9, [Computer Software]. Retrieved from https//www.jamovi.org, accessed on 19 March 2022, Sydney, Australia). Mean differences were tested with independent *t*-tests (t) when significant outliers, identified with boxplots were excluded, normal distribution of the dependent variable, tested with the Shapiro–Wilk test and homoscedasticity, tested with Levene’s test, were met. Mean differences of non-normal dependent variable data are analyzed with the Mann–Whitney U test (U). For categorical variables, a contingency table was created. To test correlations between categorical variables, the chi-square test was used. It indicates probability with which the observations of the study can be transferred to the population. A *p*-value of <0.05 was defined as significant, a value of <0.01 as very significant, and a value of <0.001 as highly significant. A significance level of *p* > 0.05 was set for hypothesis testing. Binomial logistic regression was used to determine the predicative power of the individual parameters.

## 3. Results

### 3.1. Descriptives

The collective consists of 251 free flaps which were transplanted in 115 women (45.8%) and 136 men (54.2%). The patients were 61.4 (SD = 14.9) years old on average and had a BMI of 24.6 kg/m^2^ (SD = 4.68). For detailed descriptives please see Table 1.

### 3.2. Comparison of Pre- and Postoperative Values

**Figure 1:**
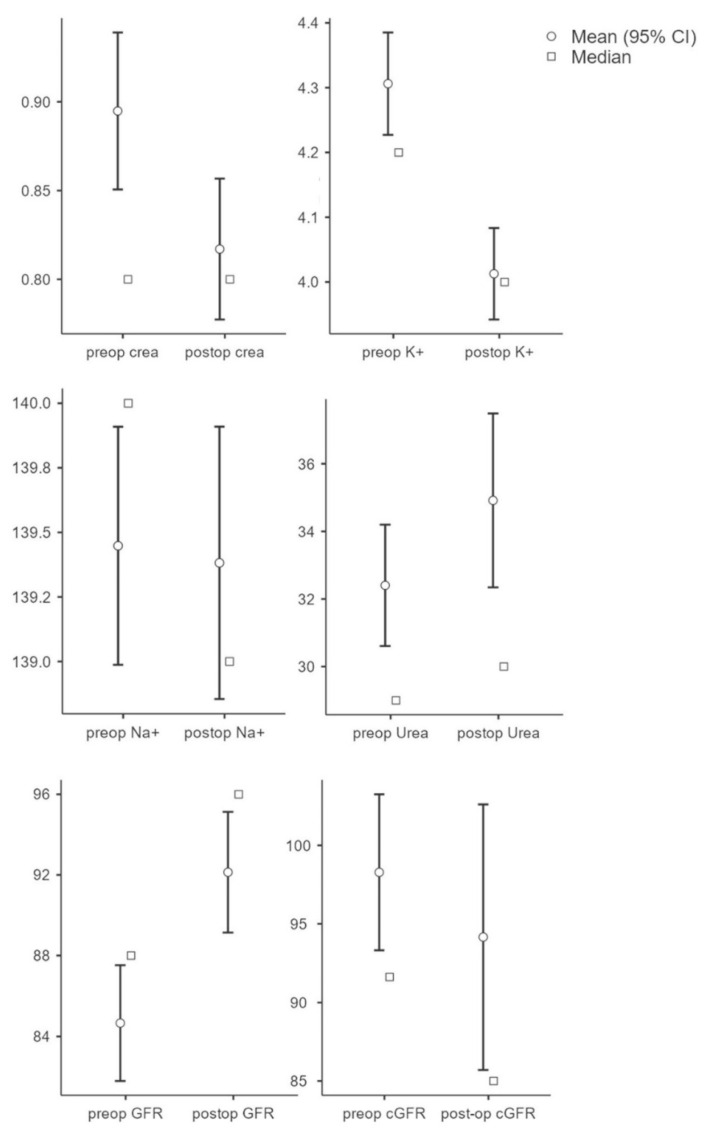
Overview of parameters (creatinine, potassium, sodium, urea, GFR, and cGRF) in pre- and postoperative comparison.

To evaluate the influence of the kidney parameters on the outcome, the individual pre- and postoperative parameters were initially evaluated and compared. The results showed that the preoperative creatinine value (M = 0.90, SD = 0.36) was significantly (t(250) = 6.64, *p* < 0.001, n = 251) higher compared to the postoperative value (M = 0.82, SD = 0.32). Potassium values (n = 212) were significantly higher (t(211) = 5.90, *p* < 0.001) preoperatively (M = 4.31, SD = 0.59) compared to postoperatively (M = 4.01, SD = 0.52). There was no significant change in sodium (preop: M = 139.45, SD = 3.42; postop: M = 139.38, SD = 3.91; t(211) = 0.25, *p* = 0.81, n = 212) and urea values (preop: M = 32.40, SD = 14.14; postop: M = 34.92, SD = 20.24); t(237) = −1.96, *p* = 0.052, n = 238). GFR showed a significant increase postoperatively (M = 92.13, SD = 23.31) compared to preoperatively (M = 84.67, SD = 22.37; t(250) = −7.53; *p* < 0.001; n = 233). There was no significant difference in GFR (preop: M = 98.28, SD = 40.19; postop: M = 94.16, SD = 68.36); t(251) = 0.95; *p* = 0.345, n = 251) (see Figure 1).

### 3.3. Predictive Power of the Parameters

We performed binomial logistic regression to determine the impact of preoperative serum creatinine and predict the likelihood of complications occurring. The binomial logistic regression model was statistically significant for potassium (χ^2^(1) = 4.47, *p* = 0.035, Nagelkerke’s R^2^ = 0.002) and sodium (χ^2^(1) = 7.2, *p* = 0.007, Nagelkerke’s R^2^ = 0.004). The overall percentage of accuracy in classification was 58.9%, with a sensitivity of 13.3% and a specificity of 92.9% for potassium, and an overall percentage of accuracy in classification was 61.4%, with a sensitivity of 27.6% and a specificity of 86.5%. Increasing potassium levels are more likely to cause complications. As sodium levels rise, complications are less likely to occur. The binomial logistic regression models of the other parameters were not statistically significant. All model coefficients, predictive measures, and odds can be found in Table 2. A binomial logistic regression was also performed to determine the effect of the parameters and predict the likelihood of contracting mortality. The binomial logistic regression models were not statistically significant. All model coefficients, predictive measures, and odds can be found in Table 1. Another binomial logistic regression was conducted to determine the effect of preoperative creatinine and predict the likelihood of contracting a failure of the graft. The model was not statistically significant, χ^2^(1) = 0.101, *p* = 0.075, resulting in a small amount of explained variance (Backhaus et al., 2003), as shown by Nagelkerke’s R^2^ = 0.001. The overall percentage of accuracy in classification was 65.3%, with a sensitivity of 100% and a specificity of 0%. For the other parameters, a non-statistically significant model was found with respect to graft failure: Preoperative Potassium (χ^2^(1) = 2.46, *p* = 0.117, Nagelkerke’s R^2^ = 0.001. Overall percentage of accuracy in classification was 66.3%, with a sensitivity of 2.3% and a specificity of 99,4%.); Preoperative sodium (χ^2^(1) = 1.48, *p* = 0.224, Nagelkerke’s R^2^ = 0.001. Overall percentage of accuracy in classification was 65.9%, with a sensitivity of 100% and a specificity of 0%.); Preoperative Urea (χ^2^(1) = 0.082, *p* = 0.774, Nagelkerke’s R^2^ = 0.000. Overall percentage of accuracy in classification was 65.9%, with a sensitivity of 100% and a specificity of 0%.); Preoperative GFR (χ^2^(1) = 0.114, *p* = 0.736, Nagelkerke’s R^2^ = 0.000. Overall percentage of accuracy in classification was 66%, with a sensitivity of 100% and a specificity of 0%.); Preoperative cGFR (χ^2^(1) = 1.19, *p* = 0.276, Nagelkerke’s R^2^ = 0.006. Overall percentage of accuracy in classification was 80.5%, with a sensitivity of 100% and a specificity of 0%.).

### 3.4. Cut-off Creatinine 1.2 mg/dL

Creatinine clearance is the most common clinical clearance method for assessing renal function and is an important diagnostic tool for detecting renal insufficiency. Above 1.2 mg/dL, serum creatinine is considered elevated and is used as a cut-off in this study. In 225 cases, the serum creatinine was below 1.2 mg/dL, in 26 cases, above 1.2 mg/dL. In the first group (serum creatinine < 1.2 mg/dL) there were 106 (47.1%) female and 119 (52.9%) male patients. These patients were 60.3 (SD = 14.9) years old on average and showed a BMI of 24.5 kg/m^2^ (SD = 4.63). In 67.1% (n = 151), a microvascular graft was necessary due to tumor disease, in 22.2% (n = 50) for secondary reconstruction, in 7.1% (n = 16) due to osteoradionecrosis, in 1.3% (n = 3) due to osteomyelitis, and in 0.9% (n = 2) due to MONJ. In three cases (1.3%) no further classification was made, e.g., for a basal cell carcinoma. The second group (serum creatinine > 1.2 mg/dL) consists of 9 female and 17 male patients. These patients were 71.2 (SD = 11.0) years old and showed a BMI of 25.0 kg/m^2^ (SD = 5.09). Compared to the first group, there was a significant difference regarding the age of the patients (U = 1678; *p* < 0.001; r = 0.427). In 80.7% (n = 21) a microvascular graft was necessary due to tumor disease, in 7.7% (n = 2) for secondary reconstruction, and in 3.9% (n = 1), each due to osteoradionecrosis and osteomyelitis. In one case (3.9%) no further classification was made. No patient suffered from an MONJ. The parameters, whose role as possible preoperative risk factors should be evaluated, show the following in the first group. The preoperative potassium was 4.29 mmol/L (SD = 0.58), the preoperative sodium was 139.3 mmol/L (SD = 3.45), and the preoperative urea was 30.75 mol/L (SD = 12.6). The estimated glomerular filtration rate was 89.5 mL/min (SD = 18.8) and 104.2 mL/min (SD = 37.7) using the Cockcroft–Gault formula.

**Figure 2:**
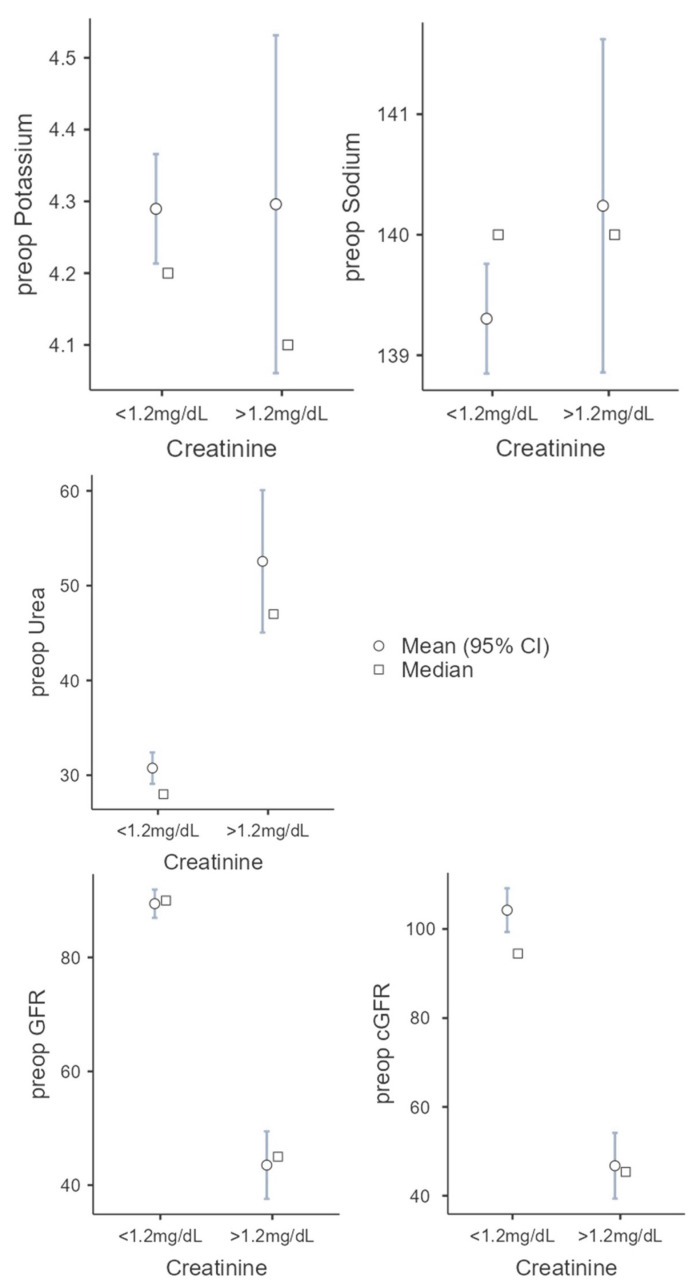
Comparison of the two groups (serum creatine cut-off 1.2 mg/dL) regarding the following parameters: potassium, sodium, urea, GFR, and cGRF.

The following values were found in the second group: preoperative potassium 4.3 mmol/L (SD = 0.6), preoperative sodium 140.2 mmol/L (SD = 3.5), preoperative urea 52.56 mol/l (SD = 19.2), GFR. 43.52 mL/min (SD = 15.2), and cGFR 46.75 mL/min (SD = 19.3). With increased creatine, there were significant differences in preoperative GFR (t(242) = 11.7987; *p* < 0.001; Cohen’s d = 2.491) and preoperative cGFR (U = 338; *p* < 0.001; r = 0.885) compared to the first group, as expected. The second group showed a higher level of urea with 52.56 mol/L (SD = 19.2; U = 764; *p* < 0.001; r = 0.724). There was no statistical significance between the two groups regarding the preoperative potassium (U = 2668; *p* = 0.780; r = 0.034) and preoperative sodium (U = 2390; *p* = 0.266; r = 0.135) (see Figure 2).

### 3.5. Creatinine

Comparing the complications of the graft, there were not significantly more complications in the first group (30.7%, n = 69/125) than in the other group (34.6%, n = 9/26; Χ2 (1) = 0.170; *p* = 0.680; Cramers’V = 0.026). In 20% (1st: n = 45/125), respectively, in 19.2% (2nd: n = 5/26), of the cases a revision of the anastomosis was necessary. No significant difference was found with Χ2 ((1) = 0.009; *p* = 0.926; Cramers’V = 0.006). Regarding the failure of the graft, there was no significant difference between the two groups (1st: 20.0% (n = 45/255); 2nd 15.4% (n = 4/26); Χ2 (1) = 0.316; *p* = 0.574; Cramers’V = 0.035). With 40.0% (n = 90/225), there were significantly fewer complications in the first group than in the second group with 61.5% (n = 16/26; Χ2 (1) = 4.43; *p* = 0.035; Cramers’V = 0.133). In the first group 2.7% (n = 6/219) of patients and 38.5% (n = 10/26) of the patients in the second group died. We found a highly significant correlation between the serum creatinine and mortality using the Chi-square test (Χ2 (1) = 27.9; *p* < 0.001; Cramers’V = 0.334)). In addition to transplant complications, the other postoperative complications showed the following: in the first group, no complications occurred in 50.2% (n = 113), delirium occurred in 7.1% (n = 16), respiratory complications in 6.7% (n = 15), and AKI in 1.2% (n = 4). In the second group, no complications occurred in 30.8% (n = 8), delirium occurred in 30.8% (n = 8), respiratory complications in 23.1% (n = 6), sepsis in 15.4%, and AKI in 19.2% (n = 5).

## 4. Discussion

Successful microvascular reconstruction is characterized by a satisfactory healing process, a low complication rate, and the prevention of further surgical interventions [5,7,8,14,15,24]. Comparing pre- and postoperative values, there was a significant decrease in potassium and creatinine levels and a significant increase in GFR. This might be attributed to the optimized circulatory volume and oxygen therapy during an operation lasting several hours. Looking closely at the electrolyte changes in relation to the complication rate, it can be seen that complications were more likely to occur as potassium levels increased. As sodium levels increase, the complication rate decreases. Disturbances of the electrolyte balance always have an influence on the nutritive supply of the various organ systems. If the normal electrolyte values cannot be maintained, the supply, especially of the vulnerable transplants, deteriorates. Existing research indicates an association between the serum creatinine level with infection of the surgical site after free flap reconstruction [25]. The impairment of the renal function seems to be predictive for a potential failure of the graft [26,27], especially with additional diabetes mellitus and peripheral vascular disease [13,28]. The results demonstrate that the complication rate is significantly increased at a serum creatinine level above 1.2mg/dL, but this does not refer to graft complications. Studies showed that patients with serum creatinine levels >1.28 mg dL^−1^ had significantly higher free flap transfer complication rates than those with serum creatinine levels <1.28 mg dL^−1^ (*p* = 0.038) [29]. Compared to existing research, we showed that other complications, such as respiratory problems, acute renal failure, delirium, and death, were increased in patients with elevated creatinine levels [30]. The highly significant correlation between serum creatinine and mortality in this study was demonstrated, as well as in cardiovascular [31] and abdominal surgeries [32]. A preoperative value indicating impaired renal function, such as creatinine levels above 1.2 mg dL^−1^, was not found to be an individual risk factor for the occurrence of graft failure in this patient population. Interestingly, patients with impaired renal function especially showed significantly improved renal values after several hours of surgery. This might be attributed to the optimized circulatory, volume, and oxygen therapy during an operation lasting several hours. Usually, these operations do not lead to severe blood pressure and circulation complications, nor to significant blood loss, which are known risk factors for the development of acute renal failure. Laboratory parameters can also recover in patients with impaired renal function who receive intensive medical care during the surgical procedure, as well as inpatient care under optimized conditions. The resulting normative electrolytes do not represent an individual risk for predicting graft failure. Despite this, the accurate and early identification of patients at risk of AKI and the targeted delivery of treatment protocols are of benefit. For example, optimization of hemodynamics and volume, close monitoring of renal function, and avoidance of nephrotoxins are recommended by Kidney Disease: Improving Global Outcomes (KDIGO) [33,34]. Nevertheless, there are only a few studies on the relationship between preoperative creatine levels and postoperative complications in microvascular grafts in the current literature [30].

The validity of a retrospective data analysis is limited even if the quality and validity criteria are fulfilled. The limiting factors include the fact that the available diagnoses cannot be retrospectively verified regarding their etiology and completeness. Furthermore, missing data cannot be determined retrospectively; this also applies to incomplete data sets.

## 5. Conclusions

A preoperative value indicative of impaired renal function, such as creatinine levels, GFR, or electrolytes, did not prove to be a solitary risk factor for the occurrence of graft failure in this patient population. Nevertheless, increased renal parameters are associated with increased incidence of serious complications and should therefore be considered in indication, preoperative planning, and future research.

## Figures and Tables

**Table 1:** Descriptives.

	Total
Age	
Years (Mean ±SD)	61.4 ± 14.9
Gender	
Male	136 (54.2%)
Female	115 (45.8.6%)
Diagnosis	
tumor disease	68.5% (n = 172)
secondary reconstruction	20.7% (n = 52)
osteoradionecrosis,	6.8% (n = 17)
osteomyelitis	1.2% (n = 3)
MONJ	1.2% (n = 3)
no further classification	1.6% (n = 4)
Flap design	
Fascio-cutaneous, musculocutaneous	13.1% (n = 33) 24.3% (n = 61)
osteo-musculocutaneous	62.5% (n = 157)
Anastomosis	
arterial anastomosis end-to-end end-to-side venous anastomosis	98.8% (n = 248) 1.2% (n = 3)
end-to-end	80.5% (n = 202)
end-to-side	19.5% (n = 50)
Complications	
Pedicle thrombosis	31.1% (n = 78)
Ischemia	46.8% (n = 36)
Congestion Graft failure Mortality	53.2% (n = 41) 19.5% (n = 49) 5.2% (n = 13)
Hospital stay (days)	
Mean ± SD	37.4 ± 33.3
Maximum	7
Minimum	246

**Table 2:** Overview Model Coefficients: Models for predictability of the preoperative parameters (creatinine, potassium, sodium, urea, GFR, and cGRF) of complications, mortality, and failure of the graft. * Note. The cut-off value is set to 0.5.

Model Coefficients—Complications								Model Fit Measures					Predictive Measures *		
						95% Confidence Interval				Overall Model Test					
Predictor	Estimate	SE	Z	*p*	Odds Ratio	Lower	Upper	Model	R^2^_N_	χ^2^	df	*p*	Accuracy	Specificity	Sensitivity
Intercept	0.321	0.381	0.843	0.399	1.379	0.653	2.91								
preop creatinine	−0.68	0.411	−1.66	0.098	0.507	0.227	1.13	1	0.0163	3.07	1	0.08	0.566	0.986	0.00926
Intercept	−2.378	1.031	−2.31	0.021	0.0927	0.0123	0.7								
preop potassium	0.485	0.238	2.04	0.042	1.6242	1.0182	2.591	1	0.0242	4.47	1	0.04	0.589	0.929	0.133
Intercept	13.843	5.3817	2.57	0.01	1,030,000	26.99	39,200,000,000.00								
preop sodium	−0.101	0.0386	−2.63	0.009	0.904	0.838	0.975	1	0.0387	7.2	1	0.01	0.614	0.865	0.276
Intercept	0.0429	0.3201	0.134	0.893	1.044	0.557	1.95								
preop urea	−0.0103	0.009	−1.15	0.252	0.99	0.972	1.01	1	0.00735	1.35	1	0.25	0.577	1	0.00952
Intercept	−1.2114	0.514	−2.36	0.018	0.298	0.109	0.816								
preop GFR	0.0107	0.0058	1.85	0.064	1.011	0.999	1.022	1	0.0192	3.52	1	0.06	0.582	0.929	0.115
Intercept	−0.56957	0.3394	−1.68	0.093	0.566	0.291	1.1								
preop cGFR	0.00293	0.0032	0.921	0.357	1.003	0.997	1.01	1	0.00455	0.852	1	0.36	0.574	0.986	0.0278
**Model Coefficients—Mortality**								**Model Fit Measures**					**Predictive Measures**		
						**95% Confidence** **Interval**				**Overall Model Test**					
**Predictor**	**Estimate**	**SE**	**Z**	***p***	**Odds** **ratio**	**Lower**	**Upper**	**Model**	**R^2^_N_**	**χ^2^**	**df**	***p***	**Accuracy**	**Specificity**	**Sensitivity**
Intercept	−1.026	0.357	−2.87	0.004	0.358	0.178	0.722								
preop creatinine	0.396	0.366	1.08	0.279	1.485	0.725	3.041	1	0.00645	1.17	1	0.28	0.665	1	0.0118
Intercept	−2.023	1.006	−2.01	0.044	0.132	0.0184	0.95								
preop potassium	0.313	0.231	1.36	0.175	1.368	0.8698	2.151	1	0.0104	1.86	1	0.17	0.663	0.994	0.012
Intercept	6.0173	5.3743	1.12	0.263	410.452	0.0109	15,400,000.00								
preop sodium	−0.048	0.0386	−1.24	0.213	0.953	0.8837	1.03	1	0.00868	1.55	1	0.21	0.663	1	0
Intercept	−1.1456	0.329	−3.48	<0 .001	0.318	0.167	0.606								
preop urea	0.0141	0.0089	1.59	0.113	1.014	0.997	1.032	1	0.0141	2.51	1	0.11	0.663	0.982	0.0361
Intercept	−0.38779	0.5113	−0.76	0.448	0.679	0.249	1.85								
preop GFR	−0.00347	0.0059	−0.59	0.554	0.997	0.985	1.01	1	0.00199	0.35	1	0.55	0.664	1	0
Intercept	−0.32829	0.3592	−0.91	0.361	0.72	0.356	1.46								
preop cGFR	−0.0035	0.0035	−1.01	0.311	0.997	0.99	1	1	0.00582	1.06	1	0.3	0.661	1	0
**Model Coefficients—Failure of the graft**								**Model Fit Measures**					**Predictive Measures**		
						**95% Confidence** **Interval**				**Overall Model Test**					
**Predictor**	**Estimate**	**SE**	**Z**	***p***	**Odds ratio**	**Lower**	**Upper**	**Model**	**R^2^_N_**	**χ^2^**	**df**	***p***	**Accuracy**	**Specificity**	**Sensitivity**
Intercept	−0.527	0.364	−1.45	0.148	0.591	0.289	1.2								
preop creatinine	−0.12	0.381	−0.32	0.752	0.887	0.421	1.87	1	0.00056	0.101	1	0.75	0.653	1	0
Intercept	−2.21	1.012	−2.18	0.029	0.11	0.0151	0.798								
preop potassium	0.361	0.232	1.55	0.121	1.434	0.9096	2.262	1	0.0138	2.46	1	0.12	0.663	0.994	0.0238
Intercept	5.8768	5.362	1.1	0.273	356.649	0.0097	13,100,000.00								
preop sodium	−0.0469	0.0385	−1.22	0.223	0.954	0.8848	1.03	1	0.0083	1.48	1	0.22	0.659	1	0
Intercept	−0.74203	0.3265	−2.27	0.023	0.476	0.251	0.903								
preop urea	0.00258	0.009	0.287	0.774	1.003	0.985	1.02	1	0.00046	0.082	1	0.77	0.659	1	0
Intercept	−0.83085	0.5188	−1.6	0.109	0.436	0.158	1.2								
preop GFR	0.00198	0.0059	0.337	0.736	1.002	0.99	1.01	1	0.00065	0.114	1	0.74	0.66	1	0
Intercept	−0.27449	0.3576	−0.77	0.443	0.76	0.377	1.53								
preop cGFR	−0.00369	0.0035	−1.07	0.284	0.996	0.99	1	1	0.0065	1.19	1	0.28	0.653	1	0

## Data Availability

The data presented in this study are available on request from the corresponding author. The data are not publicly available due to privacy regulations.
